# Central line-associated bloodstream infections in patients with COVID-19

**DOI:** 10.1590/1518-8345.7007.4236

**Published:** 2024-07-29

**Authors:** Nicole Caetano Acosta, Rodrigo do Nascimento Ceratti, Marina Scherer Santos, Simone de Souza Fantin, Fernanda Fuzinatto, Omar Pereira de Almeida Neto, Eneida Rejane Rabelo-Silva

**Affiliations:** 1 Universidade Federal do Rio Grande do Sul, Escola de Enfermagem, Porto Alegre, RS, Brazil.; 2 Universidade Federal do Rio Grande do Sul, Porto Alegre, RS, Brazil.; 3 Hospital de Clínicas de Porto Alegre, Porto Alegre, RS, Brazil.; 4 Universidade Federal de Uberlândia, Faculdade de Medicina, Uberlândia, MG, Brazil.

**Keywords:** COVID-19, Central Venous Catheterization, Sepsis, Prone Position, Mechanical Ventilation, ICU

## Abstract

**Objective:**

to investigate the association between central line-associated bloodstream infections and clinical and care variables of intensive care unit patients with COVID-19 hospitalized at a reference public health institution.

**Method:**

a case-control study.

**Results:**

the study sample consisted of 70 patients diagnosed with central line-associated bloodstream infections (case group) and 70 non-infected patients (control group). Most patients were male, with mean age of 57.93±13.93 years old and provided with a double lumen catheter. Median time of central line-associated bloodstream infections onset was 11 (8-18) days. Longer time on mechanical ventilation ( *P* =0.014; OR: 1.79; 95% CI: 0.91-3.51) and prone position ( *P* =0.017; OR: 2.41; 95% CI: 1.22-4.81) were associated with central line-associated bloodstream infections onset.

**Conclusion:**

longer time on invasive mechanical ventilation and prone position contributed to central line-associated bloodstream infections onset in COVID-19 patients.

## Highlights

**(1)** During ICU hospitalization, the central venous catheter (CVC) is essential.

**(2)** Patients with COVID-19 in the ICU should be observed for infection.

**(3)** Longer time on invasive mechanical ventilation and prone are related factors.

## Introduction

 Patients with risk factors for severe COVID-19 such as age over 60 years old, multiple comorbidities and no or delayed vaccinations, may experience hypoxemic respiratory failure, acute respiratory distress syndrome (ARDS) or septic shock, requiring admission into an intensive care unit (ICU) ^(^ ^1^ ^)^ . The care provided to severe ICU patients consists of managing COVID-19-related morbidities by administering antithrombotics and cytokine inhibitors, and by preventing ICU-related complications, such as ventilator-associated pneumonia and central line-associated bloodstream infections (CLABSI) ^(^ ^1^ ^-^ ^3^ ^)^ . 

 During ICU hospitalization, central venous catheterization (CVC) is essential for sedoanalgesia, infusion of vasoactive drugs and large volumes, antibiotics, repeated blood collection, and other infusions incompatible with peripheral veins ^(^ ^4^ ^-^ ^6^ ^)^ . Adult ICU patients, as a priority, usually receive double lumen catheters, inserted in jugular or femoral veins, to allow for infusion therapy ^(^ ^3^ ^-^ ^7^ ^)^ . 

 ICU patients with COVID-19 are at an increased risk of CLABSI onset, due to a high production of oral and tracheal secretions, clinical severity, corticosteroid therapy, invasive procedures performed during treatment, and high workload, which can hinder proper CVC insertion and manipulation practices ^(^ ^8^ ^)^ . Other complications such as catheter displacement, bleeding, thrombosis and obstruction can also occur ^(^ ^7^ ^-^ ^8^ ^)^ . 

Despite numerous publications on the COVID-19 infection, research data and clinical results concerning CLABSI are still in development, as well as their association with clinical variables and care processes. Studying CLABSI in ICU patients with COVID-19 is essential to design institutional strategies, improve care, and achieve positive clinical outcomes. Based on this gap in the literature, this study investigated the association between CLABSI and clinical and care variables of ICU patients with COVID-19 hospitalized at a reference public health institution. Its results allow us to identify variables associated with unfavorable clinical outcomes, findings that can lead to changes in care processes and contribute to positive and safer results for patients and healthcare teams.

## Method

### Study design

Case-control study with retrospective data.

### Context

 Research was conducted with patients admitted to the COVID-19 Intensive Care Unit of the public hospital *Hospital de Clínicas de Porto Alegre* (HCPA), Rio Grande do Sul, Brazil, from March 2020 to May 2021. 

### Sample

 Sample size was estimated using the PSS Health tool ^(^ ^9^ ^)^ , online version, to detect differences in the average time of mechanical ventilation between the case and control groups, with a difference of 4.35 days as relevant to the study. Considering an 80% power, 5% significance level, and standard deviation of 7.27 days, obtained from a pilot study data bank with 20 subjects (10 in the case group and 10 in the control group), the total sample size estimation reached 88 subjects, 44 in each group. Adding 10% for possible losses, the sample size should be at least 98 (49 in each group). Finally, we included 70 patients in each group (n=140), based on a report by the HCPA’s Infection Control Committee. 

Eligible participants were adult patients admitted to HCPA’s COVID-19 ICU using a CVC, monitored by electronic medical records available in the institutional software (AGHUse) and of the hospital’s Vascular Access Program (VAP) database, from CVC insertion in the COVID-19 ICU until its removal.

### Eligibility criteria

Case Group: Adults (age > 18 years) with a medical diagnosis of COVID-19, admitted to an intensive care unit, using a CVC, monitored by electronic medical records in the AGHUse, with central line-associated bloodstream infection during ICU stay confirmed by blood culture (BC) positive with a pathogen unrelated to other infectious focus or two or more positive BCs by skin contaminants collected at different times associated with clinical symptoms (fever >38°C, chills, hypotension).

Control Group: Adults (age > 18 years) with a medical diagnosis of COVID-19, admitted to an intensive care unit, using a CVC, monitored by electronic medical records in the AGHUse, without positive CLABSI by blood culture during ICU stay.

### Data collection

 Based on variables available in the HCPA’s AGHUse software and VAP database, we developed an instrument containing demographic and clinical variables, variables related to current hospitalization and CVC-related management variables. Patients were monitored from insertion to removal, with 24 hours added to identify possible catheter-related infections ^(^ ^10^ ^)^ . 

### Data analysis

Data were managed on the REDCap platform and then imported into the Statistical Package for the Social Sciences (SPSS) program, version 21.0. Exploratory (descriptive) data analysis was based on estimating simple absolute frequencies and percentages for categorical variables. Quantitative variables were expressed as mean and standard deviation or median and interquartile range, according to data distribution. Associations between primary bloodstream infection and CVC-related clinical and care variables were estimated by Pearson’s chi-square test and Odds Ratio (OR). A two-sided P <0.05 was considered statistically significant.

### Ethical aspects

This study was approved by the National Health Council and by the HCPA’s Research Ethics Committee under Certificate of Presentation of Ethical Appreciation number 09223119.4.0000.5327, and followed the Guidelines and Regulatory Standards for Research involving human beings.

## Results

### Demographic and clinical characteristics

The study sample included 140 patients with CVC admitted to the COVID-19 ICU, 70 diagnosed with CLABSI (case group) and 70 without infection (control group).

 When analyzing the demographic and clinical characteristics ( Table 1 ), we observed a higher frequency of male patients (62.9% in the case group; 57.1% in the control group). Mean age was similar in both groups (56.75±12.54 years and 59.09±15.18 years in the case and control groups, respectively). Systemic arterial hypertension (case group: 70%; control group: 60%) and diabetes (case group: 37.1%; control group: 35.7%) were the most prevalent risk factors associated with severe COVID-19 infection. Of the 140 hospitalized patients, 51 (36.4%) had thromboembolic events, mainly pulmonary thromboembolism (22.9%). 

**Table 1:** - Demographic and clinical characteristics of study participants. Porto Alegre, RS, Brazil, 2021

**Parameter**	**All** **(n=140)** **n (%)**	**Case** **(n=70)** **n (%)**	**Control** **(n=70)** **n (%)**	**P** ^*^
**Age (years)**	57.93±13.93	56.75±12.54	59.09±15.18	0.497
**Male**	84 (60)	44 (62.9)	40 (57.1)	0.605
**Risk Factors**				
Systemic Arterial Hypertension	91 (65.0)	49 (70.0)	42 (60.0)	0.288
Age > 60	63 (45)	29 (41.4)	34 (48.6)	0.497
Diabetes	51 (36.4)	26 (37.1)	25 (35.7)	1.000
Obesity (BM ^I^ ^†^ > 30kg/m²)	24 (17.1)	15 (21.4)	9 (12.9)	0.262
Previous smoking	19 (13.6)	8 (11.4)	11 (15.7)	0.622
Cardiovascular diseases	9 (6.4)	6 (8.7)	3 (4.3)	0.493
Chronic Obstructive Pulmonary Disease	9 (6.4)	3 (4.3)	6 (8.6)	0.493
Moderate/severe asthma	8 (5.7)	5 (7.1)	3 (4.3)	0.718
Chronic Kidney Disease (grade 3, 4, 5)	8 (5.7)	3 (4.3)	5 (7.1)	0.718
Immunosuppression	7 (5.0)	3 (4.3)	4 (5.7)	1.000
Cancer	6 (4.3)	1 (1.4)	5 (7.1)	0.209
Anemia	5 (3.6)	4 (5.7)	1 (1.4)	0.366
Active smoking	4 (2.9)	2 (2.9)	2 (2.9)	1.000
None	13 (9.3)	6 (8.6)	7 (10)	1.000
Thromboembolic events	51 (36.4)	27 (38.6)	24 (34.3)	0.725
Pulmonary thromboembolism	32 (22.9)	16 (22.9)	16 (22.9)	1.000

* Chi-square test;

† BMI = Body Mass Index

### Main invasive and non-invasive procedures during ICU stay

 All patients in the case group received invasive mechanical ventilation, compared to 98.6% in the control group. Interventions such as hemodialysis (37.1%), insertion of other CVCs (36.4%) and non-invasive mechanical ventilation (32.1%) were also widely implemented. Patients in the case group spent more time on mechanical ventilation, 20 (13.7 – 27.2) days compared to 15 (12 – 23) days in the control group (OR: 1.79; 95% CI: 0.91–3.51), showing a significant difference in the association with CLABSI ( *P* =0.014). 

 We also observed a significant difference between groups ( *P* =0.017) regarding the association between CLABSI and patients who required a pronation maneuver during COVID-19 ICU stay (OR: 2.41; 95% CI: 1.22–4.81). However, case and control groups showed no significant difference for the association between the number of pronation maneuvers and pronation time ( *P* =0.062 and *P* =0.226, respectively). 

 Of the 140 patients, nine (6.4%) underwent tracheostomy, seven (10%) in the case group and two (2.9%) in the control group ( *P* =0.165). Table 2 shows other data related to the procedures carried out on the sample. 

**Table 2:** - Invasive and non-invasive procedures carried out on patients admitted to the COVID-19 Intensive Care Unit. Porto Alegre, RS, Brazil, 2021

**Variables**	**All** **(n=140)** **n (%)**	**Case** **(n=70)** **n (%)**	**Control** **(n=70)** **n (%)**	**P** ^*^
Invasive mechanical ventilation	139 (99.3)	70 (100.0)	69 (98.6)	1.000
Hemodialysis	52 (37.1)	25 (35.7)	27 (38.6)	0.861
Another concomitant CVC ^†^	51 (36.4)	24 (34.3)	27 (38.6)	0.725
Pronation	79 (56.4)	47 (67.1)	32 (45.7)	0.017
Noninvasive mechanical ventilation	45 (32.1)	23 (32.9)	22 (31.4)	1.000
High-flow nasal catheter	36 (25.7)	22 (31.4)	14 (20.0)	0.175
Tracheostomy	9 (6.4)	7 (10.0)	2 (2.9)	0.165
ECMO ^‡^	4 (2.9)	4 (5.7)	0 (0.0)	0.12
Invasive mechanical ventilation time	-	20 (13.7 – 27.2)	15 (12 – 23)	0.014

* Chi-square test;

† CVC = Central venous catheter;

‡ ECMO = Extracorporeal membrane oxygenation

Average time to the occurrence of CLABSI was 11 (8–18) days.

### Characteristics of central access devices

 In both the case and control groups, the double-lumen catheter was the most commonly used (case: 97.1%; control: 95.7%), followed by the single-lumen catheter (2.1%) and the triple-lumen catheter (1.4%). Regarding insertion site, most catheters were inserted into the internal jugular veins (case: 74.2%; control: 85.7%), followed by femoral (case: 14.3%; control: 8.6%), subclavian (case: 8.6%; control: 4.3%), and axillary veins (case: 2.9%; control: 1.4%). When associating CLABSI with CVC puncture site and number of lumens, we found no statistically significant difference ( *P* =0.701 and *P* =1.000, respectively). 

The most prevalent indications for CVC insertion were sedoanalgesia (case: 98.6%; control: 97.1%), use of vasoactive drugs (case: 77.1%; control: 68.6%), and large-volume infusion (case: 11.4%; control: 20%). None of these variables differed statistically between groups.

### CVC-related complications

 Insertion bleeding (case: 12.9%; control: 10%) and obstruction of one or more lumens (case: 10%; control: 8.6%) were the main complications registered in the medical records. Doppler ultrasound confirmed the occurrence of vessel thrombosis in six patients and accidental traction in two others. Table 3 shows that none of these variables differed statistically between the groups. 

**Table 3:** - Central venous catheter-related complications in patients admitted to the COVID-19 Intensive Care Unit. Porto Alegre, RS, Brazil, 2021

**Parameter**	**All** **(n=140)** **n (%)**	**Case** **(n=70)** **n (%)**	**Control** **(n=70)** **n (%)**	**p** ^*^
Bleeding	18 (12.9)	7 (10)	11 (15.7)	0.450
Obstruction	14 (10)	6 (8.6)	8 (11.4)	0.779
Hyperemia	10 (7.1)	5 (7.1)	5 (7.1)	1.000
Thrombosis	6 (4.3)	4 (5.7)	2 (2.9)	0.681
Accidental traction	2 (1.4)	2 (2.9)	0 (0)	0.496

* Chi-square test

The main reasons for catheter removal were CLABSI in the case group (75.7%) and death in the control group (37.1%).

 Of the 70 CLABSI cases, 61.4% showed growth of Gram-positive bacteria and 38.6% of Gram-negative bacteria in blood culture. The most frequent infecting germs were bacteria from the coagulase-negative *Staphylococcus* group (41.4%). 

Median length of catheter use in all patients was 13 (10–18) days. We estimated similar values in the case and control groups ‒ 12.5 (9–19) and 14 (10–17) days, respectively ‒, and found no significant association with infection onset.

## Discussion

 To the best of our knowledge, this is the first study to investigate the association between CLABSI onset and clinical and care variables in COVID-19 patients admitted to the ICU. CLABSI onset was associated with length of mechanical ventilation ( *P* =0.014) and prone position ( *P* =0.017). 

 Several clinical complications require ICU treatment associated with ventilatory support. A multicenter cohort study conducted in Spain showed that, of the 667 patients included, only 165 (24%) used a high-flow nasal cannula, whereas 494 (74%) required invasive mechanical ventilation, as in our study ^(^ ^11^ ^)^ . 

 In addition to endotracheal tubes, the use of other invasive devices exponentially increases the risk of healthcare-related infections. A previously published retrospective analysis found that invasive mechanical ventilator-associated pneumonia and CLABSI onset increased as hospitalization days were prolonged (ventilator-associated pneumonia: 9.8% on day 5 to 58.8% on day 25; CLABSI: 0.9% on day 5 to 5.0% on day 25) ^(^ ^12^ ^)^ . Moreover, the length of mechanical ventilation seems to be related to CLABSI incidence in an ICU environment. Although our study found an average infection-free CVC time of 11 days (8–18), an investigation conducted in an adult ICU showed an average of 5.5 days ^(^ ^13^ ^)^ . These findings can be attributed to the clinical severity imposed by COVID-19 compared to other infectious diseases. 

 Besides invasive procedures, other non-invasive care practices are carried out for the clinical management of COVID-19. Studies indicate that applying the prone position technique to intubated patients with severe acute respiratory syndrome (SARS) and COVID-19 improves oxygenation and decreases mortality ^(^ ^14^ ^-^ ^16^ ^)^ . 

 Our results show an association between CLABSI onset and patient prone position; however, the lack of current studies that could justify this association limits us from extrapolating these results. Considering the clinical complexity and difficult management of ICU COVID-19 patients, the high load of vesicants and intravenous drugs administered during treatment and the vast number of invasive procedures associated with skin injuries that can occur due to prone position may explain the findings. Another factor to consider when discussing CLABSI and prone position is the difficulty of monitoring the insertion site of the central venous catheter and its connections, as well as the proper maintenance of dressings, which can be impaired by exposure to intrinsic (oral, tracheal, subcutaneous) and extrinsic (bed humidity, etc.) fluids during long pronation periods ^(^ ^8^ ^)^ . 

 Research recommends that the puncture site and the number of catheter lumens be evaluated according to the proposed therapy in order to reduce infusion therapy-related risks. Regarding COVID-19 specifically, international studies suggest that the choice of puncture site should be in the infraclavicular region to reduce exposure to oral and tracheal fluids, avoiding catheter-related infections; however, these sites increase the risk of pulmonary complications of mechanical ventilation ^(^ ^7^ ^-^ ^8^ ^)^ . An US cohort study observed that the risk of CLABSI increases proportionally to the number of lumens in the device ^(^ ^17^ ^)^ . Our study found that the majority of catheters were double lumen (96.4%) inserted into the jugular (80%). Based on the data collected, it was impossible to associate the number of lumens with CLABSI, as the choice of a double-lumen catheter was similar in both groups to support the proposed infusion therapy. 

 CLABSI occurs mainly by extraluminal colonization ‒ which occurs in the first two weeks due to contamination during catheter insertion ‒ and by intraluminal colonization ‒ which occurs after two weeks of stay, mostly due to inadequate handling techniques ^(^ ^18^ ^)^ . Our results show that the average time to infection was 11 (8–18) days, proving that excessive manipulation of catheter connections over time tends to favor their contamination, as well as the failure to adopt good practices. 

 Several studies have shown the prevalence of CLABSI caused by Gram-negative organisms, with coagulase-negative *Staphylococcus* being the main germ ^(^ ^19^ ^-^ ^20^ ^)^ . These findings corroborate our study, as bacteria from the coagulase-negative *Staphylococcus* group predominated in the blood cultures (41.4%), and infections may be associated with catheter handling or site colonization during insertion. 

 In addition to care, CVC complications may be related to clinical factors inherent to COVID-19, since extreme elevations of D-dimers, thrombocytopenia, decreased fibrinogen and prolonged prothrombin time occur, associated with an increase in thromboembolic events. Anticoagulants are often used for prophylaxis in critically ill patients, which may increase the risk of bleeding ^(^ ^21^ ^)^ . In our study, apart from infectious complications, the most frequent events were insertion bleeding (18/12.9%) and obstruction of one or more lumens (14/10%). 

Our study results show the importance of adopting good practices in CVC insertion, maintenance, and management to avoid complications that could lead to unfavorable outcomes to patients. Identifying the factors associated with infection onset, such as invasive mechanical ventilation and prone position, allows teams to review protocols for the adoption of good practices, care checklists and ongoing training, aimed at reducing these unfavorable outcomes.

As this is a study with retrospective data collection, there may be limitations in finding reliable data in medical records, due to unsatisfactory recording. COVID-19 was an emerging disease, and these data may still be considered insufficient regarding vascular access, especially in associations between clinical and care variables.

## Conclusion

Data analysis showed that longer length of invasive mechanical ventilation and prone position were associated with CLABSI onset in COVID-19 patients.
