# Supplementary material for: Central line-associated bloodstream infections in patients with COVID-19
Source: Rev Lat Am Enfermagem. 2024 Jul 29;32:e4236. doi: 10.1590/1518-8345.7007.4236 (PMC11295261; doi:10.1590/1518-8345.7007.4236)
Supplement: Translation

# Translation: Infecciones de la circulación sanguínea relacionadas con catéter venoso central en pacientes con COVID-19

**DOI:** 10.1590/1518-8345.7007.4235

**Authors:** Nicole Caetano Acosta, Rodrigo do Nascimento Ceratti, Marina Scherer Santos, Simone de Souza Fantin, Fernanda Fuzinatto, Omar Pereira de Almeida Neto, Eneida Rejane Rabelo-Silva

**Affiliations:** 1 Universidade Federal do Rio Grande do Sul, Escola de Enfermagem, Porto Alegre, RS, Brasil.; 2 Universidade Federal do Rio Grande do Sul, Porto Alegre, RS, Brasil.; 3 Hospital de Clínicas de Porto Alegre, Porto Alegre, RS, Brasil.; 4 Universidade Federal de Uberlândia, Faculdade de Medicina, Uberlândia, MG, Brasil.

**Keywords:** COVID-19, Cateterismo Venoso Central, Sepsis, Posición Prona, Respiración Artificial, Unidades de Cuidados Intensivos

## Abstract

**Objetivo:**

investigar la asociación entre infecciones de la circulación sanguínea relacionadas con catéter venoso central y variables clínicas y asistenciales de pacientes con COVID-19 ingresados en la unidad de cuidados intensivos de una institución pública de salud de referencia.

**Método:**

un estudio caso-control.

**Resultados:**

la muestra del estudio estuvo compuesta por 70 pacientes con diagnóstico de infección de la circulación sanguínea relacionada con catéter venoso central (grupo caso) y 70 pacientes no infectados (grupo control). La mayoría de los pacientes eran del sexo masculino, con edad media de 57,93±13,93 años y provistos de catéter de doble luz. El tiempo medio de aparición de las infecciones del torrente sanguíneo asociadas a catéter venoso central fue de 11 (8-18) días. Un mayor tiempo en ventilación mecánica ( *P* =0,014; RP: 1,79; IC 95%: 0,91-3,51) y en posición de decúbito prono ( *P* =0,017; RP: 2,41; IC del 95 %: 1,22-4,81) se asociaron con la aparición de infecciones de la circulación sanguínea relacionadas con catéter venoso central.

**Conclusión:**

un tiempo más prolongado con ventilación mecánica invasiva y posición de decúbito prono contribuyeron a la aparición de infecciones de la circulación sanguínea relacionadas con catéter venoso central en pacientes con COVID-19.

## Destacados

**(1)** Durante la hospitalización en UCI, el catéter venoso central (CVC) es fundamental.

**(2)** Los pacientes con COVID-19 en la UCI deben ser observados para detectar infecciones.

**(3)** Mayor tiempo en ventilación mecánica invasiva y posición de decúbito prono son factores relacionados.

## Introducción

 Pacientes con factores de riesgo de COVID-19 grave, como edad mayor de 60 años, múltiples comorbilidades y vacunación nula o retrasada, pueden experimentar insuficiencia respiratoria hipoxémica, síndrome de dificultad respiratoria aguda (SDRA) o choque séptico, requiriendo ingreso en una unidad de cuidados intensivos (UCI) ^(^ ^1^ ^)^ . La atención brindada a los pacientes graves de la UCI consiste en el manejo de las morbilidades relacionadas con la COVID-19 mediante la administración de antitrombóticos e inhibidores de citoquinas, y la prevención de complicaciones relacionadas con la UCI, como la neumonía asociada a la ventilación mecánica y las infecciones de la circulación sanguínea relacionadas con catéter venoso central ^(^ ^1^ ^-^ ^3^ ^)^ . 

 Durante la hospitalización en UCI, el cateterismo venoso central es fundamental para sedoanalgesia, infusión de fármacos vasoactivos y grandes volúmenes, antibióticos, extracciones repetidas de sangre y otras infusiones incompatibles con las venas periféricas ^(^ ^4^ ^-^ ^6^ ^)^ . Los pacientes adultos de la UCI, como prioridad, suelen recibir catéteres de doble luz, insertados en las venas yugulares o femorales, para permitir la terapia de infusión ^(^ ^3^ ^-^ ^7^ ^)^ . 

 Los pacientes de UCI con COVID-19 tienen mayor riesgo de infecciones de la circulación sanguínea relacionadas con catéter venoso central, debido a la alta producción de secreciones orales y traqueales, la gravedad clínica, la terapia con corticosteroides, los procedimientos invasivos realizados durante el tratamiento y la alta carga de trabajo, que pueden dificultar prácticas adecuadas de inserción y manipulación del catéter venoso central (CVC) ^(^ ^8^ ^)^ . También pueden ocurrir otras complicaciones, como desplazamiento del catéter, sangrado, trombosis y obstrucción ^(^ ^7^ ^-^ ^8^ ^)^ . 

A pesar de numerosas publicaciones sobre la infección por COVID-19, aún se encuentran en desarrollo datos de investigaciones y resultados clínicos sobre infecciones de la circulación sanguínea relacionadas con catéter venoso central, así como su asociación con variables clínicas y procesos asistenciales. Estudiar infecciones de la circulación sanguínea relacionadas con catéter venoso central en pacientes de UCI con COVID-19 es esencial para diseñar estrategias institucionales, mejorar el cuidado y lograr resultados clínicos positivos. A partir de ese vacío en la literatura, este estudio investigó la asociación entre infecciones de la circulación sanguínea relacionadas con catéter venoso central y variables clínicas y asistenciales de pacientes con COVID-19 ingresados en la UCI de una institución pública de salud de referencia. Sus resultados permiten identificar variables asociadas a resultados clínicos desfavorables, hallazgos que pueden conducir a cambios en los procesos de cuidado y contribuir a resultados positivos y más seguros para los pacientes y los equipos de salud.

## Método

### Diseño del estudio

Se trata de un estudio caso-control con datos retrospectivos.

### Contexto

 La investigación se realizó con pacientes ingresados en la Unidad de Cuidados Intensivos COVID-19 del hospital público *Hospital de Clínicas de Porto Alegre* (HCPA), Rio Grande do Sul, Brasil, de marzo de 2020 a mayo de 2021. 

### Muestra

 El tamaño de la muestra se estimó mediante la herramienta *PSS Health* ^(^ ^9^ ^)^ , versión *online* , para detectar diferencias en el tiempo promedio de ventilación mecánica entre los grupos caso y control, siendo una diferencia de 4,35 días relevante para el estudio. Considerando un poder del 80%, un nivel de significancia del 5% y una desviación estándar de 7,27 días, obtenidos de un banco de datos de un estudio piloto con 20 individuos (10 en el grupo caso y 10 en el grupo control), la estimación del tamaño total de la muestra alcanzó 88 individuos, 44 en cada grupo. Sumando un 10% por posibles pérdidas, el tamaño de la muestra debería ser de al menos 98 (49 en cada grupo). Finalmente, incluimos 70 pacientes en cada grupo (n = 140), según un informe de la Comisión de Control de Infecciones del HCPA. 

 Los participantes elegibles fueron pacientes adultos ingresados en la UCI COVID-19 del HCPA utilizando un CVC, monitoreados por registros médicos electrónicos disponibles en el *software* institucional (AGHUse) y de la base de datos del Programa de Acceso Vascular (PAV) del hospital, desde la inserción del CVC en la UCI COVID-19 hasta su eliminación. 

### Criterios de selección

Grupo caso: Adultos (edad > 18 años) con diagnóstico médico de COVID-19, ingresados en una unidad de cuidados intensivos, utilizando un CVC, monitoreados mediante registros médicos electrónicos en el AGHUse, con infección de la circulación sanguínea relacionada con catéter venoso central durante la hospitalización en la UCI confirmada por hemocultivo (HC) positivo con un patógeno no relacionado con otro foco infeccioso o dos o más HC positivos por contaminantes cutáneos recolectados en diferentes momentos asociados con síntomas clínicos (fiebre >38°C, escalofríos, hipotensión).

Grupo Control: Adultos (edad > 18 años) con diagnóstico médico de COVID-19, ingresados en unidad de cuidados intensivos, mediante CVC, monitoreados por registros médicos electrónicos en el AGHUse, sin infección de la circulación sanguínea relacionada con catéter venoso central positiva por hemocultivo durante la hospitalización en UCI.

### Recolección de datos

 Con base en las variables disponibles en el *software* AGHUse y la base de datos PAV del HCPA, desarrollamos un instrumento que contiene variables demográficas y clínicas, variables relacionadas con la hospitalización actual y variables de manejo relacionadas con catéter. Los pacientes fueron monitoreados desde la inserción hasta la extracción, y se agregaron 24 horas para identificar posibles infecciones relacionadas con el catéter ^(^ ^10^ ^)^ . 

### Análisis de los datos

 Los datos fueron gestionados en la plataforma REDCap y luego importados al programa *Statistical Package for the Social Sciences* (SPSS), versión 21.0. El análisis de datos exploratorio (descriptivo) se basó en la estimación de frecuencias absolutas simples y porcentajes para variables categóricas. Las variables cuantitativas se expresaron como media y desviación estándar o mediana y rango intercuartílico, según la distribución de los datos. Las asociaciones entre la infección de la circulación sanguínea y las variables clínicas y asistenciales relacionadas con el CVC se estimaron mediante la prueba de chi-cuadrado de Pearson y la Razón de posibilidades (RP). Una P bilateral <0,05 se consideró estadísticamente significativa. 

### Aspectos éticos

Este estudio fue aprobado por el Consejo Nacional de Salud y por el Comité de Ética en Investigación del HCPA bajo Certificado de Presentación de Apreciación Ética número 09223119.4.0000.5327, y siguió las Directrices y Normas Reguladoras de Investigaciones con seres humanos.

## Resultados

### Características demográficas y clínicas

La muestra del estudio incluyó a 140 pacientes con CVC ingresados en la UCI COVID-19, 70 con diagnóstico de infección de la circulación sanguínea relacionada con catéter venoso central (grupo caso) y 70 sin infección (grupo control).

 Al analizar las características demográficas y clínicas ( Tabla 1 ), observamos una mayor frecuencia de pacientes del sexo masculino (62,9% en el grupo caso; 57,1% en el grupo control). La edad media fue similar en ambos grupos (56,75 ± 12,54 años y 59,09 ± 15,18 años en los grupos caso y control, respectivamente). La hipertensión arterial sistémica (grupo caso: 70%; grupo control: 60%) y la diabetes (grupo caso: 37,1%; grupo control: 35,7%) fueron los factores de riesgo más prevalentes asociados con la infección grave por COVID-19. De los 140 pacientes hospitalizados, 51 (36,4%) tuvieron eventos tromboembólicos, principalmente tromboembolismo pulmonar (22,9%). 

**Tabla 1:** - Características demográficas y clínicas de los participantes del estudio. Porto Alegre, RS, Brasil, 2021

**Parámetro**	**Todos** **(n=140)** **n (%)**	**Caso** **(n=70)** **n (%)**	**Control** **(n=70)** **n (%)**	**P** ^*^
**Edad (años)**	57,93±13,93	56,75±12,54	59,09±15,18	0,497
**Sexo masculino**	84 (60)	44 (62,9)	40 (57,1)	0,605
**Factores de riesgo**				
Hipertensión arterial sistémica	91 (65,0)	49 (70,0)	42 (60,0)	0,288
Edad > 60	63 (45)	29 (41,4)	34 (48,6)	0,497
Diabetes	51 (36,4)	26 (37,1)	25 (35,7)	1,000
Obesidad (IMC ^†^ > 30kg/m²)	24 (17,1)	15 (21,4)	9 (12,9)	0,262
Tabaquismo previo	19 (13,6)	8 (11,4)	11 (15,7)	0,622
Enfermedades cardiovasculares	9 (6,4)	6 (8,7)	3 (4,3)	0,493
Enfermedad Pulmonar Obstructiva Crónica	9 (6,4)	3 (4,3)	6 (8,6)	0,493
Asma moderada/grave	8 (5,7)	5 (7,1)	3 (4,3)	0,718
Enfermedad renal crónica (etapas 3, 4, 5)	8 (5,7)	3 (4,3)	5 (7,1)	0,718
Inmunosupresión	7 (5,0)	3 (4,3)	4 (5,7)	1,000
Cáncer	6 (4,3)	1 (1,4)	5 (7,1)	0,209
Anemia	5 (3,6)	4 (5,7)	1 (1,4)	0,366
Tabaquismo activo	4 (2,9)	2 (2,9)	2 (2,9)	1,000
Ninguno	13 (9,3)	6 (8,6)	7 (10)	1,000
Eventos tromboembólicos	51 (36,4)	27 (38,6)	24 (34,3)	0,725
Tromboembolismo pulmonar	32 (22,9)	16 (22,9)	16 (22,9)	1,000

* Prueba de chi-cuadrado;

† IMC = Índice de Masa Corporal

### Principales procedimientos invasivos y no invasivos durante la hospitalización en UCI

 Todos los pacientes del grupo caso recibieron ventilación mecánica invasiva, frente al 98,6% del grupo control. También se implementaron ampliamente intervenciones como la hemodiálisis (37,1%), la inserción de otros CVC (36,4%) y la ventilación mecánica no invasiva (32,1%). Los pacientes del grupo caso pasaron más tiempo con ventilación mecánica, 20 (13,7 – 27,2) días en comparación con 15 (12 – 23) días en el grupo control (RP: 1,79; IC 95%: 0,91–3,51), lo que muestra una diferencia significativa en la asociación con infecciones de la circulación sanguínea relacionadas con catéter venoso central ( *P* =0,014). 

 También observamos una diferencia significativa entre los grupos ( *P* =0,017) con respecto a la asociación entre infecciones de la circulación sanguínea relacionadas con catéter venoso central y pacientes que requirieron una maniobra de pronación durante la hospitalización en la UCI COVID-19 (RP: 2,41; IC 95%: 1,22–4,81). Sin embargo, los grupos caso y control no mostraron diferencias significativas en la asociación entre el número de maniobras de pronación y el tiempo de pronación ( *P* =0,062 y *P* =0,226, respectivamente). 

 De los 140 pacientes, nueve (6,4%) fueron sometidos a traqueotomía, siete (10%) en el grupo caso y dos (2,9%) en el grupo control ( *P* =0,165). En la Tabla 2 se muestran otros datos relacionados con los procedimientos realizados en la muestra. 

**Tabla 2:** - Procedimientos invasivos y no invasivos realizados en pacientes ingresados en la Unidad de Cuidados Intensivos COVID-19. Porto Alegre, RS, Brasil, 2021

**Variables**	**Todos** **(n=140)** **n (%)**	**Caso** **(n=70)** **n (%)**	**Control** **(n=70)** **n (%)**	**P** ^*^
Ventilación mecánica invasiva	139 (99,3)	70 (100,0)	69 (98,6)	1,000
Hemodiálisis	52 (37,1)	25 (35,7)	27 (38,6)	0,861
Otro CVC ^†^ concomitante	51 (36,4)	24 (34,3)	27 (38,6)	0,725
Decúbito prono	79 (56,4)	47 (67,1)	32 (45,7)	0,017
Ventilación mecánica no invasiva	45 (32,1)	23 (32,9)	22 (31,4)	1,000
Catéter nasal de alto flujo	36 (25,7)	22 (31,4)	14 (20,0)	0,175
Traqueotomía	9 (6,4)	7 (10,0)	2 (2,9)	0,165
ECMO ^‡^	4 (2,9)	4 (5,7)	0 (0,0)	0,12
Tiempo de ventilación mecánica invasiva	-	20 (13,7 – 27,2)	15 (12 – 23)	0,014

* Prueba de chi-cuadrado;

† CVC = Catéter venoso central;

‡ ECMO = Oxigenación por membrana extracorpórea

El tiempo promedio hasta la aparición de infección de la circulación sanguínea relacionada con catéter venoso central fue de 11 (8-18) días.

### Características de los dispositivos de acceso central

 Tanto en el grupo caso como en el control, el catéter de doble luz fue el más utilizado (caso: 97,1%; control: 95,7%), seguido del catéter de una luz (2,1%) y del catéter de triple luz (1,4%). En cuanto al sitio de inserción, la mayoría de los catéteres se insertaron en la vena yugular interna (caso: 74,2%; control: 85,7%), seguida de la femoral (caso: 14,3%; control: 8,6%), subclavia (caso: 8,6%; control: 4,3%) y venas axilares (caso: 2,9%; control: 1,4%). Al asociar infecciones de la circulación sanguínea relacionadas con catéter venoso central con el sitio de punción del CVC y el número de luces, no encontramos diferencias estadísticamente significativas ( *P* =0,701 y *P* =1,000, respectivamente). 

Las indicaciones más prevalentes para la inserción de CVC fueron sedoanalgesia (caso: 98,6%; control: 97,1%), uso de fármacos vasoactivos (caso: 77,1%; control: 68,6%) e infusión de gran volumen (caso: 11,4%; control: 20%). Ninguna de estas variables difirió estadísticamente entre los grupos.

### Complicaciones relacionadas con el CVC

 El sangrado de inserción (caso: 12,9%; control: 10%) y la obstrucción de una o más luces (caso: 10%; control: 8,6%) fueron las principales complicaciones registradas en las historias clínicas. La ecografía Doppler confirmó la aparición de trombosis vascular en seis pacientes y tracción accidental en otros dos. La Tabla 3 muestra que ninguna de estas variables difirió estadísticamente entre los grupos. 

**Tabla 3:** - Complicaciones relacionadas con el catéter venoso central en pacientes ingresados en la Unidad de Cuidados Intensivos COVID-19. Porto Alegre, RS, Brasil, 2021

**Parámetro**	**Todos** **(n=140)** **n (%)**	**Caso** **(n=70)** **n (%)**	**Control** **(n=70)** **n (%)**	**p** ^*^
Sangrado	18 (12,9)	7 (10)	11 (15,7)	0,450
Obstrucción	14 (10)	6 (8,6)	8 (11,4)	0,779
Hiperemia	10 (7,1)	5 (7,1)	5 (7,1)	1,000
Trombosis	6 (4,3)	4 (5,7)	2 (2,9)	0,681
Tracción accidental	2 (1,4)	2 (2,9)	0 (0)	0,496

* Prueba de chi-cuadrado

Los principales motivos de retirada del catéter fueron infecciones de la circulación sanguínea relacionadas con catéter venoso central en el grupo caso (75,7%) y muerte en el grupo control (37,1%).

 De los 70 casos de infecciones de la circulación sanguínea relacionadas con catéter venoso central, el 61,4% presentó crecimiento de bacterias Gram positivas y el 38,6% de bacterias Gram negativas en hemocultivo. Los gérmenes infecciosos más frecuentes fueron bacterias del grupo de los *Staphylococcus* coagulasa negativo (41.4%). 

La mediana de duración del uso del catéter en todos los pacientes fue de 13 (10-18) días. Estimamos valores similares en los grupos caso y control – 12,5 (9-19) y 14 (10-17) días, respectivamente –, y no encontramos ninguna asociación significativa con la aparición de la infección.

## Discusión

 Hasta donde sabemos, este es el primer estudio que investiga la asociación entre la aparición de infecciones de la circulación sanguínea relacionadas con catéter venoso central y variables clínicas y asistenciales en pacientes con COVID-19 ingresados en UCI. El inicio de infecciones de la circulación sanguínea relacionadas con catéter venoso central se asoció con la duración de la ventilación mecánica ( *P* =0,014) y la posición de decúbito prono ( *P* =0,017). 

 Varias complicaciones clínicas requieren tratamiento en UCI asociado con soporte ventilatorio. Un estudio de cohorte multicéntrico realizado en España demostró que, de los 667 pacientes incluidos, sólo 165 (24%) utilizaron cánula nasal de alto flujo, mientras que 494 (74%) requirieron ventilación mecánica invasiva, al igual que en nuestro estudio ^(^ ^11^ ^)^ . 

 Además de los tubos endotraqueales, el uso de otros dispositivos invasivos aumenta exponencialmente el riesgo de infecciones relacionadas con la atención de salud. Un análisis retrospectivo publicado anteriormente encontró que la neumonía asociada a la ventilación mecánica invasiva y la aparición de infecciones de la circulación sanguínea relacionadas con catéter venoso central aumentaron a medida que se prolongaban los días de hospitalización (neumonía asociada a la ventilación: 9,8% el día 5 a 58,8% el día 25; ITSACC: 0,9% el día 5 a 5,0% el día 25) ^(^ ^12^ ^)^ . Asimismo, la duración de la ventilación mecánica parece estar relacionada con la incidencia de infecciones de la circulación sanguínea relacionadas con catéter venoso central en un entorno de UCI. Aunque nuestro estudio encontró un tiempo promedio de CVC libre de infección de 11 días (8-18), una investigación realizada en una UCI de adultos mostró un promedio de 5,5 días ^(^ ^13^ ^)^ . Estos hallazgos pueden atribuirse a la gravedad clínica impuesta por la COVID-19 en comparación con otras enfermedades infecciosas. 

 Además de los procedimientos invasivos, se llevan a cabo otras prácticas de cuidado no invasivas para el manejo clínico de la COVID-19. Estudios indican que aplicar la técnica de pronación a pacientes intubados con síndrome respiratorio agudo severo (SRAS) y COVID-19 mejora la oxigenación y disminuye la mortalidad ^(^ ^14^ ^-^ ^16^ ^)^ . 

 Nuestros resultados muestran una asociación entre el inicio de infección de la circulación sanguínea relacionada con catéter venoso central y la posición de decúbito prono del paciente; sin embargo, la falta de estudios actuales que puedan justificar esta asociación nos limita a la hora de extrapolar estos resultados. Teniendo en cuenta la complejidad clínica y el difícil manejo de los pacientes de la UCI con COVID-19, la alta carga de vesicantes y medicamentos intravenosos administrados durante el tratamiento y la gran cantidad de procedimientos invasivos asociados con lesiones en la piel que pueden ocurrir debido a la posición boca abajo pueden explicar los hallazgos. Otro factor a considerar cuando se habla de infecciones de la circulación sanguínea relacionadas con catéter venoso central y la posición de decúbito prono es la dificultad de monitorear el sitio de inserción del catéter venoso central y sus conexiones, así como el mantenimiento adecuado de los apósitos, que pueden verse afectados por la exposición a agentes intrínsecos (oral, traqueal, subcutáneo) y extrínsecos (humedad de la cama, etc.) durante períodos prolongados de pronación ^(^ ^8^ ^)^ . 

 Una investigación recomienda que el sitio de punción y la cantidad de luces del catéter se evalúen de acuerdo con la terapia propuesta para reducir los riesgos relacionados con la terapia de infusión. Respecto específicamente a la COVID-19, estudios internacionales sugieren que la elección del sitio de punción debe ser en la región infraclavicular para reducir la exposición a los fluidos orales y traqueales, evitando infecciones relacionadas con el catéter; sin embargo, estos sitios aumentan el riesgo de complicaciones pulmonares de la ventilación mecánica ^(^ ^7^ ^-^ ^8^ ^)^ . Un estudio de cohorte estadounidense observó que el riesgo de infección de la circulación sanguínea relacionada con catéter venoso central aumenta proporcionalmente al número de lúmenes del dispositivo ^(^ ^17^ ^)^ . Nuestro estudio encontró que la mayoría de los catéteres eran de doble luz (96,4%) insertados en la yugular (80%). Con base en los datos recolectados, fue imposible asociar el número de luces con infección de la circulación sanguínea, ya que la elección de catéter de doble luz fue similar en ambos grupos para apoyar la terapia de infusión propuesta. 

 Infección de la circulación sanguínea relacionada con catéter venoso central sucede principalmente por colonización extraluminal ‒ que ocurre en las primeras dos semanas debido a la contaminación durante la inserción del catéter ‒ y por colonización intraluminal ‒ que ocurre después de dos semanas de hospitalización, sobre todo debido a técnicas de manipulación inadecuadas ^(^ ^18^ ^)^ . Nuestros resultados muestran que el tiempo medio hasta la infección fue de 11 (8-18) días, lo que comprueba que la manipulación excesiva de las conexiones de los catéteres a lo largo del tiempo tiende a favorecer su contaminación, así como la falta de adopción de buenas prácticas. 

 Diversos estudios han demostrado la prevalencia de infección de la circulación sanguínea relacionada con catéter venoso central causada por organismos Gram negativos, siendo el *Staphylococcus* coagulasa negativo el principal germen ^(^ ^19^ ^-^ ^20^ ^)^ . Estos hallazgos corroboran nuestro estudio, ya que en los hemocultivos predominaron bacterias del grupo de los *Staphylococcus* coagulasa negativo (41,4%), y las infecciones pueden estar asociadas con la manipulación del catéter o la colonización del sitio durante la inserción. 

 Además de los cuidados, las complicaciones del CVC pueden estar relacionadas con factores clínicos inherentes a la COVID-19, ya que se producen elevaciones extremas de los dímeros D, trombocitopenia, disminución del fibrinógeno y tiempo de protrombina prolongado, asociados a un aumento de los eventos tromboembólicos. Los anticoagulantes se utilizan a menudo para la profilaxis en pacientes críticos, lo que puede aumentar el riesgo de hemorragia ^(^ ^21^ ^)^ . En nuestro estudio, además de las complicaciones infecciosas, los eventos más frecuentes fueron el sangrado de inserción (18/12,9%) y la obstrucción de una o más luces (14/10%). 

 Los resultados de nuestro estudio muestran la importancia de adoptar buenas prácticas en la inserción, mantenimiento y manejo del CVC para evitar complicaciones que puedan conducir a resultados desfavorables para los pacientes. La identificación de los factores asociados a la aparición de la infección, como la ventilación mecánica invasiva y la posición de decúbito prono, permite a los equipos revisar protocolos para la adopción de buenas prácticas, *checklists* de cuidados y capacitación continua, orientadas a reducir esos resultados desfavorables. 

Como se trata de un estudio con recolección de datos retrospectiva, puede haber limitaciones para encontrar datos confiables en los registros médicos, debido a un registro insatisfactorio. La COVID-19 era una enfermedad emergente y estos datos aún pueden considerarse insuficientes en cuanto al acceso vascular, especialmente en las asociaciones entre variables clínicas y asistenciales.

## Conclusión

El análisis de los datos mostró que una mayor duración de ventilación mecánica invasiva y posición de decúbito prono se asociaron con la aparición de infecciones de la circulación sanguínea relacionadas con catéter venoso central en pacientes con COVID-19.

# Translation: Infecção primária de corrente sanguínea relacionada a cateter venoso central em pacientes com COVID-19

**DOI:** 10.1590/1518-8345.7007.4237

**Authors:** Nicole Caetano Acosta, Rodrigo do Nascimento Ceratti, Marina Scherer Santos, Simone de Souza Fantin, Fernanda Fuzinatto, Omar Pereira de Almeida Neto, Eneida Rejane Rabelo-Silva

**Affiliations:** 1 Universidade Federal do Rio Grande do Sul, Escola de Enfermagem, Porto Alegre, RS, Brasil.; 2 Universidade Federal do Rio Grande do Sul, Porto Alegre, RS, Brasil.; 3 Hospital de Clínicas de Porto Alegre, Porto Alegre, RS, Brasil.; 4 Universidade Federal de Uberlândia, Faculdade de Medicina, Uberlândia, MG, Brasil.

**Keywords:** COVID-19, Cateterismo Venoso Central, Sepse, Decúbito Ventral, Ventilação Mecânica, UTI

## Abstract

**Objetivo:**

investigar a associação entre infecção primária de corrente sanguínea relacionada a cateter venoso central e variáveis clínicas e assistenciais de pacientes com COVID-19 internados na unidade de terapia intensiva de uma instituição pública de saúde de referência.

**Método:**

estudo caso-controle.

**Resultados:**

o estudo foi composto por 70 pacientes com diagnóstico de infecção primária de corrente sanguínea relacionada a cateter venoso central (grupo caso) e 70 pacientes sem infecção (grupo controle). Pacientes predominantemente do sexo masculino, média de idade de 57,93±13,93 anos e portadores de cateter de duplo lúmen. A mediana de tempo de ocorrência das infecções primárias de corrente sanguínea relacionadas a cateter venoso central foi de 11 (8-18) dias. Maior tempo em ventilação mecânica ( *P* =0,014; RP: 1,79; IC 95%: 0,91-3,51) e posição prona ( *P* =0,017; RP: 2,41; IC 95%: 1,22-4,81) foram associados à ocorrência de infecções primárias de corrente sanguínea relacionadas a cateter venoso central.

**Conclusão:**

maior tempo em ventilação mecânica invasiva e posição prona contribuíram para a ocorrência de infecções primárias de corrente sanguínea relacionadas a cateter venoso central em pacientes com COVID-19.

## Destaques

**(1)** Durante a internação na UTI, o cateter venoso central (CVC) é essencial.

**(2)** Pacientes com COVID-19 na UTI devem ser observados quanto a infecções.

**(3)** Maior tempo em ventilação mecânica invasiva e prona são fatores relacionados.

## Introdução

 Pacientes com fatores de risco para COVID-19 grave, como idade acima de 60 anos, múltiplas comorbidades e nenhuma vacinação ou com vacinas atrasadas, podem apresentar insuficiência respiratória hipoxêmica, síndrome do desconforto respiratório agudo (SDRA) ou choque séptico, necessitando internação em unidade de terapia intensiva (UTI) ^(^ ^1^ ^)^ . O cuidado prestado aos pacientes graves da UTI consiste no manejo das morbidades relacionadas à COVID-19, através da administração de antitrombóticos e inibidores de citocinas, e na prevenção de complicações relacionadas à UTI, como pneumonia associada à ventilação mecânica e infecções primárias de corrente sanguínea (IPCS) relacionadas a cateter venoso central (CVC) ^(^ ^1^ ^-^ ^3^ ^)^ . 

 Durante a internação na UTI, o cateterismo venoso central é essencial para sedoanalgesia, infusão de drogas vasoativas e de grandes volumes, antibióticos, coletas de sangue repetidas e outras infusões incompatíveis com veias periféricas ^(^ ^4^ ^-^ ^6^ ^)^ . Pacientes adultos internados em UTI, prioritariamente, recebem cateteres de duplo lúmen, inseridos em veias jugulares ou femorais, para permitir terapia de infusão ^(^ ^3^ ^-^ ^7^ ^)^ . 

 Pacientes de UTI com COVID-19 apresentam risco aumentado de IPCS relacionada a CVC, devido à alta produção de secreções orais e traqueais, gravidade clínica, corticoterapia, procedimentos invasivos realizados durante o tratamento e alta carga de trabalho, que podem dificultar práticas adequadas de inserção e manipulação do CVC ^(^ ^8^ ^)^ . Outras complicações como deslocamento do cateter, sangramento, trombose e obstrução também podem estar presentes ^(^ ^7^ ^-^ ^8^ ^)^ . 

Apesar das inúmeras publicações sobre a infecção por COVID-19, os dados de pesquisas e resultados relativos às IPCS relacionadas a CVC ainda estão em desenvolvimento, bem como sua associação com variáveis clínicas e processos assistenciais. Estudar IPCS relacionada a CVC em pacientes de UTI com COVID-19 é essencial para traçar estratégias institucionais, melhorar o cuidado e alcançar resultados clínicos positivos. Com base nessa lacuna da literatura, objetivou-se investigar a associação entre IPCS relacionada a CVC e variáveis clínicas e assistenciais de pacientes com COVID-19 internados na UTI de uma instituição pública de saúde de referência. Seus resultados permitem identificar variáveis associadas a desfechos clínicos desfavoráveis, achados que podem levar a mudanças nos processos de cuidado e contribuir para resultados positivos e mais seguros para pacientes e equipes de saúde.

## Método

### Delineamento do estudo

Estudo caso-controle com coleta de dados retrospectivos.

### Contexto

A pesquisa foi realizada com pacientes internados na Unidade de Terapia Intensiva COVID-19 do hospital público Hospital de Clínicas de Porto Alegre (HCPA), Rio Grande do Sul, Brasil, no período de março de 2020 a maio de 2021.

### Amostra

 O tamanho da amostra foi estimado por meio da ferramenta *PSS Health* ^(^ ^9^ ^)^ , versão *online* , para detectar diferenças no tempo médio de ventilação mecânica entre os grupos caso e controle, sendo uma diferença de 4,35 dias relevante para o estudo. Considerando um poder de 80%, nível de significância de 5% e desvio-padrão de 7,27 dias, obtidos de um banco de dados de estudo piloto com 20 indivíduos (10 no grupo caso e 10 no grupo controle), a estimativa do tamanho total da amostra atingiu 88 indivíduos, 44 em cada grupo. Somando 10% para possíveis perdas, o tamanho da amostra deveria ser de no mínimo 98 (49 em cada grupo). Por fim, incluímos 70 pacientes em cada grupo (n=140), com base em relatório da Comissão de Controle de Infecção do HCPA. 

 Os participantes elegíveis foram pacientes adultos internados na UTI COVID-19 do HCPA em uso de CVC, monitorados por prontuários eletrônicos disponíveis no *software* institucional (AGHUse) e do banco de dados do Programa de Acesso Vascular (PAV) do hospital, a partir da inserção do CVC na UTI COVID-19 até a sua remoção. 

### Critérios de seleção

Grupo Caso: Adultos (idade > 18 anos) com diagnóstico médico de COVID-19, internados em unidade de terapia intensiva, em uso de CVC, monitorados por prontuários eletrônicos no AGHuse, com infecção da corrente sanguínea relacionada a cateter venoso central durante internação na UTI confirmada por hemocultura (HC) positiva com patógeno não relacionado a outro foco infeccioso ou duas ou mais HC positivas por contaminantes cutâneos coletados em momentos diferentes associadas a sintomas clínicos (febre >38°C, calafrios, hipotensão).

Grupo Controle: Adultos (idade > 18 anos) com diagnóstico médico de COVID-19, internados em unidade de terapia intensiva, em uso de CVC, monitorados por prontuários eletrônicos no AGHuse, sem IPCS relacionada a CVC durante internação na UTI.

### Coleta de dados

 Com base nas variáveis disponíveis no *software* AGHuse e no banco de dados PAV do HCPA, desenvolvemos um instrumento contendo variáveis demográficas e clínicas, variáveis relacionadas à internação atual e variáveis de manejo relacionadas a CVC. Os pacientes foram monitorados desde a inserção até a remoção, com acréscimo de 24 horas para identificação de possíveis infecções relacionadas com o cateter ^(^ ^10^ ^)^ . 

### Análise dos dados

 Os dados foram gerenciados na plataforma REDCap e posteriormente importados para o programa *Statistical Package for the Social Science* s (SPSS), versão 21.0. A análise exploratória (descritiva) dos dados baseou-se na estimativa de frequências absolutas simples e percentuais para variáveis categóricas. As variáveis quantitativas foram expressas como média e desvio-padrão ou mediana e intervalo interquartil, conforme distribuição dos dados. As associações entre infecção primária da corrente sanguínea e variáveis clínicas e assistenciais relacionadas ao CVC foram estimadas pelo teste qui-quadrado de Pearson e pela razão de prevalência (RP). Um P bilateral <0,05 foi considerado estatisticamente significativo. 

### Aspectos éticos

Este estudo foi aprovado pelo Conselho Nacional de Saúde e pelo Comitê de Ética em Pesquisa do HCPA sob Certificado de Apresentação de Apreciação Ética número 09223119.4.0000.5327, e seguiu as Diretrizes e Normas Regulamentadoras de Pesquisas envolvendo seres humanos.

## Resultados

### Características demográficas e clínicas

A amostra do estudo incluiu 140 pacientes com CVC internados na UTI COVID-19, sendo 70 com diagnóstico de IPCS relacionada a CVC (grupo caso) e 70 sem infecção (grupo controle).

 Ao analisarmos as características demográficas e clínicas ( Tabela 1 ), observamos maior frequência de pacientes do sexo masculino (62,9% no grupo caso; 57,1% no grupo controle). A média de idade foi semelhante nos dois grupos (56,75±12,54 anos e 59,09±15,18 anos nos grupos caso e controle, respectivamente). Hipertensão arterial sistêmica (grupo caso: 70%; grupo controle: 60%) e diabetes (grupo caso: 37,1%; grupo controle: 35,7%) foram os fatores de risco mais prevalentes associados à infecção grave por COVID-19. Dos 140 pacientes internados, 51 (36,4%) apresentaram eventos tromboembólicos, principalmente tromboembolismo pulmonar (22,9%). 

**Tabela 1:** - Características demográficas e clínicas dos participantes do estudo. Porto Alegre, RS, Brasil, 2021

**Parâmetro**	**Todos** **(n=140)** **n (%)**	**Caso** **(n=70)** **n (%)**	**Controle** **(n=70)** **n (%)**	**P** ^*^
Idade (anos)	57,93±13,93	56,75±12,54	59,09±15,18	0,497
Sexo masculino	84 (60)	44 (62,9)	40 (57,1)	0,605
Fatores de risco				
Hipertensão Arterial Sistêmica	91 (65,0)	49 (70,0)	42 (60,0)	0,288
Idade > 60	63 (45)	29 (41,4)	34 (48,6)	0,497
Diabetes	51 (36,4)	26 (37,1)	25 (35,7)	1,000
Obesidade (IMC ^†^ > 30kg/m²)	24 (17,1)	15 (21,4)	9 (12,9)	0,262
Tabagismo prévio	19 (13,6)	8 (11,4)	11 (15,7)	0,622
Doenças cardiovasculares	9 (6,4)	6 (8,7)	3 (4,3)	0,493
Doença Pulmonar Obstrutiva Crônica	9 (6,4)	3 (4,3)	6 (8,6)	0,493
Asma moderada/grave	8 (5,7)	5 (7,1)	3 (4,3)	0,718
Doença Renal Crônica (estágios 3, 4, 5)	8 (5,7)	3 (4,3)	5 (7,1)	0,718
Imunossupressão	7 (5,0)	3 (4,3)	4 (5,7)	1,000
Câncer	6 (4,3)	1 (1,4)	5 (7,1)	0,209
Anemia	5 (3,6)	4 (5,7)	1 (1,4)	0,366
Tabagismo ativo	4 (2,9)	2 (2,9)	2 (2,9)	1,000
Nenhum	13 (9,3)	6 (8,6)	7 (10)	1,000
Eventos tromboembólicos	51 (36,4)	27 (38,6)	24 (34,3)	0,725
Tromboembolismo pulmonar	32 (22,9)	16 (22,9)	16 (22,9)	1,000

* Teste qui-quadrado;

† IMC = Índice de Massa Corporal

### Principais procedimentos invasivos e não invasivos durante a internação na UTI

 Todos os pacientes do grupo caso receberam ventilação mecânica invasiva, em comparação com 98,6% no grupo controle. Intervenções como hemodiálise (37,1%), inserção de outros CVC (36,4%) e ventilação mecânica não invasiva (32,1%) também foram amplamente implementadas. Os pacientes do grupo caso passaram mais tempo em ventilação mecânica, 20 (13,7 – 27,2) dias em comparação com 15 (12 – 23) dias no grupo controle (RP: 1,79; IC 95%: 0,91–3,51), mostrando diferença significativa na associação com IPCS relacionada a CVC ( *P* =0,014). 

 Também observamos uma diferença significativa entre os grupos ( *P* =0,017) com relação à associação entre IPCS relacionada a CVC e pacientes que necessitaram de manobra de pronação durante a internação na UTI COVID-19 (RP: 2,41; IC 95%: 1,22–4,81). Entretanto, os grupos caso e controle não apresentaram diferença significativa para a associação entre o número de manobras de pronação e o tempo de pronação ( *P* =0,062 e *P* =0,226, respectivamente). 

 Dos 140 pacientes, nove (6,4%) foram submetidos à traqueostomia, sete (10%) no grupo caso e dois (2,9%) no grupo controle ( *P* =0,165). A Tabela 2 apresenta outros dados relacionados aos procedimentos realizados na amostra. 

**Tabela 2:** - Procedimentos invasivos e não invasivos realizados em pacientes internados na Unidade de Terapia Intensiva COVID-19. Porto Alegre, RS, Brasil, 2021

**Variáveis**	**Todos** **(n=140)** **n (%)**	**Caso** **(n=70)** **n (%)**	**Controle** **(n=70)** **n (%)**	**P** ^*^
Ventilação mecânica invasiva	139 (99,3)	70 (100,0)	69 (98,6)	1,000
Hemodiálise	52 (37,1)	25 (35,7)	27 (38,6)	0,861
Outro CVC ^†^ concomitante	51 (36,4)	24 (34,3)	27 (38,6)	0,725
Pronação	79 (56,4)	47 (67,1)	32 (45,7)	0,017
Ventilação mecânica não invasiva	45 (32,1)	23 (32,9)	22 (31,4)	1,000
Cateter nasal de alto fluxo	36 (25,7)	22 (31,4)	14 (20,0)	0,175
Traqueostomia	9 (6,4)	7 (10,0)	2 (2,9)	0,165
ECMO ^‡^	4 (2,9)	4 (5,7)	0 (0,0)	0,12
Tempo de ventilação mecânica invasiva	-	20 (13,7 – 27,2)	15 (12 – 23)	0,014

* Teste qui-quadrado;

† CVC = Cateter venoso central;

‡ ECMO = Oxigenação por membrana extracorpórea

O tempo médio para a ocorrência de IPCS relacionada a CVC foi de 11 (8–18) dias.

### Características dos dispositivos de acesso central

 Tanto no grupo caso quanto no grupo controle, o cateter de duplo lúmen foi o mais utilizado (caso: 97,1%; controle: 95,7%), seguido pelo cateter de único lúmen (2,1%) e pelo cateter de triplo lúmen (1,4%). Quanto ao local de inserção, a maioria dos cateteres foi inserida nas veias jugulares internas (caso: 74,2%; controle: 85,7%), seguida pela femoral (caso: 14,3%; controle: 8,6%), subclávia (caso: 8,6%; controle: 4,3 %) e veias axilares (caso: 2,9%; controle: 1,4%). Ao associar IPCS relacionada a CVC com local de punção do CVC e número de lúmens, não encontramos diferença estatisticamente significativa ( *P* =0,701 e *P* =1,000, respectivamente). 

As indicações mais prevalentes para inserção de CVC foram sedoanalgesia (caso: 98,6%; controle: 97,1%), uso de drogas vasoativas (caso: 77,1%; controle: 68,6%) e infusão de grande volume (caso: 11,4%; controle: 20%). Nenhuma dessas variáveis diferiu estatisticamente entre os grupos.

### Complicações relacionadas ao CVC

 Sangramento de inserção (caso: 12,9%; controle: 10%) e obstrução de um ou mais lúmens (caso: 10%; controle: 8,6%) foram as principais complicações registradas nos prontuários. O ultrassom com Doppler confirmou a ocorrência de trombose vascular em seis pacientes e tração acidental em outros dois. A Tabela 3 mostra que nenhuma dessas variáveis diferiu estatisticamente entre os grupos. 

**Tabela 3:** - Complicações relacionadas ao cateter venoso central em pacientes internados na Unidade de Terapia Intensiva COVID-19. Porto Alegre, RS, Brasil, 2021

**Parâmetro**	**Todos** **(n=140)** **n (%)**	**Caso** **(n=70)** **n (%)**	**Controle** **(n=70)** **n (%)**	**p** ^*^
Sangramento	18 (12,9)	7 (10)	11 (15,7)	0,450
Obstrução	14 (10)	6 (8,6)	8 (11,4)	0,779
Hiperemia	10 (7,1)	5 (7,1)	5 (7,1)	1,000
Trombose	6 (4,3)	4 (5,7)	2 (2,9)	0,681
Tração acidental	2 (1,4)	2 (2,9)	0 (0)	0,496

* Teste qui-quadrado

Os principais motivos de retirada do cateter foram IPCS relacionada a CVC no grupo caso (75,7%) e óbito no grupo controle (37,1%).

 Dos 70 casos de IPCS relacionada a CVC, 61,4% apresentaram crescimento de bactérias Gram-positivas e 38,6% de bactérias Gram-negativas em hemocultura. Os germes infectantes mais frequentes foram bactérias do grupo *Staphylococcus* coagulase negativo (41,4%). 

O tempo médio de uso do cateter em todos os pacientes foi de 13 (10–18) dias. Estimamos valores semelhantes nos grupos caso e controle – 12,5 (9–19) e 14 (10–17) dias, respectivamente –, e não encontramos associação significativa com o surgimento da infecção.

## Discussão

 Até onde sabemos, este é o primeiro estudo a investigar a associação entre o aparecimento de IPCS relacionada a CVC e variáveis clínicas e assistenciais em pacientes com COVID-19 internados em UTI. O início da IPCS relacionada a CVC foi associado ao tempo de ventilação mecânica ( *P* =0,014) e à posição prona ( *P* =0,017). 

 Diversas complicações clínicas necessitam de tratamento em UTI associado ao suporte ventilatório. Um estudo de coorte multicêntrico realizado na Espanha mostrou que, dos 667 pacientes incluídos, apenas 165 (24%) usaram cânula nasal de alto fluxo, enquanto 494 (74%) necessitaram de ventilação mecânica invasiva, como em nosso estudo ^(^ ^11^ ^)^ . 

 Além dos tubos endotraqueais, o uso de outros dispositivos invasivos aumenta exponencialmente o risco de infecções relacionadas à assistência à saúde. Uma análise retrospectiva publicada anteriormente descobriu que a pneumonia associada à ventilação mecânica invasiva e o aparecimento de IPCS relacionada a CVC aumentaram à medida que os dias de hospitalização foram prolongados (pneumonia associada à ventilação mecânica: 9,8% no dia 5 a 58,8% no dia 25; IPCS relacionada a CVC: 0,9% no dia 5 a 5,0% no dia 25) ^(^ ^12^ ^)^ . Além disso, a duração da ventilação mecânica parece estar relacionada à incidência de IPCS relacionada a CVC em ambiente de UTI. Embora nosso estudo tenha encontrado um tempo médio de CVC livre de infecção de 11 dias (8–18), uma pesquisa realizada em uma UTI de adultos mostrou uma média de 5,5 dias ^(^ ^13^ ^)^ . Esses achados podem ser atribuídos à gravidade clínica imposta pela COVID-19 em comparação com outras doenças infecciosas. 

 Além dos procedimentos invasivos, outras práticas de cuidado não invasivas são realizadas para o manejo clínico da COVID-19. Estudos indicam que a aplicação da técnica de pronação em pacientes intubados com síndrome respiratória aguda grave (SRAG) e COVID-19 melhora a oxigenação e diminui a mortalidade ^(^ ^14^ ^-^ ^16^ ^)^ . 

 Nossos resultados mostram uma associação entre o início de IPCS relacionada a CVC e a posição prona do paciente; entretanto, a falta de estudos atuais que possam justificar essa associação nos impede de extrapolar esses resultados. Considerando a complexidade clínica e o difícil manejo dos pacientes com COVID-19 na UTI, a alta carga de vesicantes e medicamentos intravenosos administrados durante o tratamento e o grande número de procedimentos invasivos associados a lesões de pele que podem ocorrer devido à posição prona podem explicar os achados. Outro fator a ser considerado quando se discute IPCS relacionada a CVC e posição prona é a dificuldade de monitoramento do local de inserção do cateter venoso central e suas conexões, bem como a manutenção adequada dos curativos, que pode ser prejudicada pela exposição a agentes intrínsecos (oral, traqueal, subcutâneo) e extrínsecos (umidade do leito, etc.) durante longos períodos de pronação ^(^ ^8^ ^)^ . 

 Uma pesquisa recomenda que o local da punção e o número de lúmens do cateter sejam avaliados de acordo com a terapia proposta, a fim de reduzir os riscos relacionados à terapia infusional. Especificamente com relação à COVID-19, estudos internacionais sugerem que a escolha do local de punção deve ser na região infraclavicular para reduzir a exposição a fluidos orais e traqueais, evitando infecções relacionadas ao cateter; no entanto, esses locais aumentam o risco de complicações pulmonares da ventilação mecânica ^(^ ^7^ ^-^ ^8^ ^)^ . Um estudo de coorte nos EUA observou que o risco de IPCS relacionada a CVC aumenta proporcionalmente ao número de lúmens no dispositivo ^(^ ^17^ ^)^ . Nosso estudo constatou que a maioria dos cateteres eram de duplo lúmen (96,4%) inseridos na jugular (80%). Com base nos dados coletados, não foi possível associar o número de lúmens à IPCS relacionada a CVC, pois a escolha de cateter de duplo lúmen foi semelhante nos dois grupos para apoiar a terapia infusional proposta. 

 A IPCS relacionada a CVC acontece principalmente por colonização extraluminal - que ocorre nas primeiras duas semanas devido à contaminação durante a inserção do cateter - e por colonização intraluminal - que ocorre após duas semanas de internação, sobretudo devido a técnicas de manuseio inadequadas ^(^ ^18^ ^)^ . Nossos resultados mostram que o tempo médio para infecção foi de 11 (8–18) dias, comprovando que a manipulação excessiva das conexões dos cateteres ao longo do tempo tende a favorecer a sua contaminação, bem como a não adoção de boas práticas. 

 Vários estudos têm demonstrado a prevalência de IPCS relacionada a CVC causada por organismos Gram-negativos, sendo o *Staphylococcus* coagulase negativo o principal germe ^(^ ^19^ ^-^ ^20^ ^)^ . Esses achados corroboram nosso estudo, pois as bactérias do grupo *Staphylococcus* coagulase negativo predominaram nas hemoculturas (41,4%), e as infecções podem estar associadas ao manuseio do cateter ou à colonização do local durante a inserção. 

 Além dos cuidados, as complicações do CVC podem estar relacionadas com fatores clínicos inerentes à COVID-19, uma vez que ocorrem elevações extremas de dímeros D, trombocitopenia, diminuição do fibrinogênio e prolongamento do tempo de protrombina, associados ao aumento de eventos tromboembólicos. Os anticoagulantes são frequentemente usados para profilaxia em pacientes gravemente enfermos, o que pode aumentar o risco de sangramento ^(^ ^21^ ^)^ . Em nosso estudo, além das complicações infecciosas, os eventos mais frequentes foram sangramento de inserção (18/12,9%) e obstrução de um ou mais lúmens (14/10%). 

 Os resultados do nosso estudo mostram a importância da adoção de boas práticas na inserção, manutenção e manejo do CVC para evitar complicações que possam levar a resultados desfavoráveis aos pacientes. A identificação dos fatores associados ao aparecimento da infecção, como ventilação mecânica invasiva e posição prona, permite que as equipes revisem protocolos para a adoção de boas práticas, *checklists* de cuidados e treinamentos contínuos, visando à redução desses desfechos desfavoráveis. 

Por se tratar de um estudo com coleta de dados retrospectiva, pode haver limitações na localização de dados confiáveis em prontuários, devido a um registro insatisfatório. A COVID-19 foi uma doença emergente, e esses dados ainda podem ser considerados insuficientes no que diz respeito ao acesso vascular, especialmente nas associações entre variáveis clínicas e assistenciais.

## Conclusão

A análise dos dados mostrou que maior tempo de ventilação mecânica invasiva e posição prona foram associados ao aparecimento de IPCS relacionada a CVC em pacientes com COVID-19.
